# Intragastric Balloon as a First Step Before Metabolic Bariatric Surgery in Patients with BMI ≥ 50 kg/m^2^: are the Results After Balloon Related to Global Outcomes After Surgery?

**DOI:** 10.1007/s11695-024-07418-8

**Published:** 2024-07-23

**Authors:** André Costa Pinho, Alexandra Luís Manco, Marco Silva, Hugo Santos Sousa, Fernando Resende, John Preto, Eduardo Lima da Costa, CRI-O Group

**Affiliations:** 1 Integrated Responsibility Center for Obesity (CRI-O), São João Local Health Unit, Porto, Portugal; 2 Faculty of Medicine, University of Porto, Alameda Professor Hernâni Monteiro, 4200-319 Porto, Portugal

**Keywords:** Intragastric balloon, Obesity, BMI above 50, Metabolic and bariatric surgery, Complications

## Abstract

**Introduction:**

Patients with body mass index (BMI) ≥ 50 kg/m^2^, classified with obesity class IV/V, require complex treatments. Intragastric balloon (IGB) is a possible treatment before metabolic bariatric surgery (MBS) that may reduce peri-operative complications. This study evaluates IGB outcomes and complications before MBS in patients with Obesity IV/V, and subsequent MBS results, regarding weight loss and comorbidity resolution.

**Methods:**

Retrospective cohort study of all patients with BMI above 50 kg/m^2^ submitted to IGB before MBS between 2009 and 2023 in a high-volume center. Variables analyzed included weight loss after IGB and MBS, IGB complications, and comorbidity resolution. Suboptimal clinical responses were defined as %TWL < 5% for IGB, %TWL < 20% for MBS, and %TWL < 25% or BMI ≥ 35 kg/m^2^ for IGB + MBS**.**

**Results:**

Seventy-four patients (mean BMI 58.8 ± 8 kg/m^2^) were included. After IGB, the mean %TWL was 14.2 ± 8.5%, with a 21.6% complication rate, predominantly nausea and vomiting, and one death. Suboptimal clinical response of IGB affected 13.5% of patients, and 5.4% required early removal. Two years after MBS, the mean %TWL was 38.2 ± 11.6%, mainly due to MBS, yet approximately one-third of %TWL was attributed to IGB. No correlation was found between IGB and MBS outcomes. At 2-year follow-up, 45.1% patients had %TWL ≥ 25 and BMI < 35 kg/m^2^.

**Conclusion:**

The IGB is a treatment option before MBS in patients with Obesity Class IV/V, with acceptable weight loss outcomes but not infrequent complications. A multidisciplinary approach is mandatory, and all treatments must be considered in this difficult subset of patients.

**Graphical Abstract:**

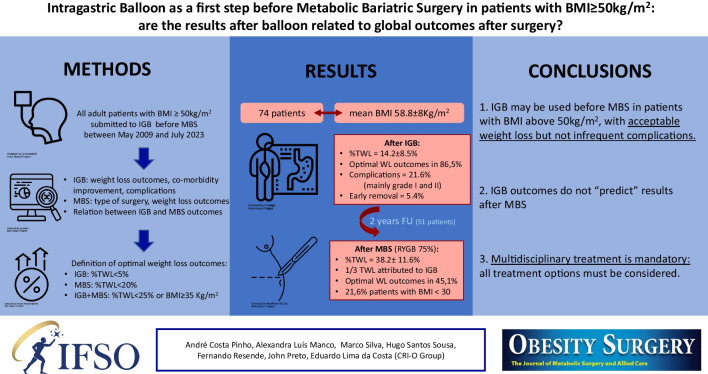

## Introduction

Obesity is a chronic disease with a complex pathophysiology that is associated to chronic low-grade inflammation and immune dysfunction, causing metabolic disturbances [1]. Individuals with obesity have an increased risk of developing conditions such as arterial hypertension (HT), obstructive sleep apnea (OSA), type 2 diabetes mellitus (DM2), and certain types of cancer [1–4]. The prevalence of obesity has surged over the years, constituting a pandemic that not only diminishes quality of life but also life expectancy [2].

Patients with body mass index (BMI) above 50 kg/m^2^, commonly classified with Obesity Class IV and V, present a severe form of obesity. Treating this condition poses several challenges regarding risks and benefits, and the optimal therapeutic approach varies depending on individual factors [5]. Metabolic bariatric surgery (MBS) is the preferred treatment for these patients, historically driven by the necessity for aggressive interventions. Various MBS options, including biliopancreatic diversion, Roux-en-Y gastric bypass (RYGB), sleeve gastrectomy (SG), single anastomosis duodenal-ileal bypass with sleeve (SADIS), and one anastomosis gastric bypass (OAGB), have been proposed [6, 7]. The incidence of peri-operative complications is higher in individuals with Obesity IV/V, compared to other obesity classes, since patients with significant visceral obesity will present challenges during intubation and mechanical ventilation [8, 9], and surgical difficulties may arise related to enlarged liver and exposure issues. These patients may also present more severe comorbidities.

Two-stage strategies have been purported to mitigate such risks, assess efficacy, and evaluate patient adherence to different treatments. The intragastric balloon (IGB) is a temporary, mechanical technique involving endoscopic placement of a balloon, inflated with air or fluid, in the patient’s stomach, for several months [10]. The balloon occupies a significant portion of the gastric volume, leading to delayed gastric emptying and reducing the urge to eat [9, 11]. While IGB as a standalone therapy yields modest long-term results, it can be used as a “neoadjuvant” therapy for MBS [12], especially in patients with high risk for medical or surgical complications, such as patients with Obesity IV/V.

Most IGB carry inherent risks, including respiratory complications due to the invasive nature of the procedure and the need for general anesthesia. Adverse events such as nausea, abdominal pain, and gastroesophageal reflux disease (GERD) may also arise. New devices were developed to mitigate these complications by eliminating the need for endoscopy during placement and removal. Furthermore, as outcomes of new anti-obesity medication continues to improve, it is crucial to reassess the role of IGB in obesity treatment, particularly as a bridge to MBS in patients with high BMI, to minimize overall complications and risks.

The main objective of this study was to evaluate the outcomes and complications of IGB as the initial step before MBS in the treatment of patients with BMI ≥ 50 kg/m^2^. Additionally, as a secondary objective, we assessed whether the results of MBS were influenced by previous IGB, namely weight loss outcomes and comorbidities resolution.

## Materials and Methods

### Study Design

We performed a retrospective cohort study of all adult patients (18 to 65 years) with BMI ≥ 50 kg/m^2^ submitted to IGB placement before MBS between May 2009 and July 2023 in a high-volume obesity center. The study was approved by the local ethics committee (416/2023). Patients submitted to revisional MBS (namely after adjustable gastric band) or undergoing other medical treatments for obesity were not excluded.

In our hospital, we consider IGB before MBS in patients with BMI ≥ 50 kg/m^2^ and high-risk features. Per protocol, all patients before IGB placement are evaluated by a multidisciplinary team, including a bariatric surgeon, an endocrinologist, a nutritionist, and a psychiatrist, to consider peri and post-operative risks. This evaluation includes abdominal ultrasonography, extensive blood and urine analysis, and upper GI endoscopy to detect potential contraindications such as active peptic ulcers, grade C–D esophagitis, and large hiatal hernias. Additional tests are frequently required. The anesthesiologist determines the best location for the procedure based on patient’s ASA classification: ASA II patients undergo IGB placement and removal in the gastroenterology department, staying for observation for 4–6 h as outpatients, whether ASA III patients require the procedure to be performed in the operating room, with at least 24 h of monitored care. Balloon volumes range from 500 to 800 ml, determined by stomach size. Patients receive instructions for a 1-month liquid diet and follow-up evaluations are performed at 2 weeks, 3 months, and 5 months post-procedure. In patients undergoing MBS after IGB, the balloon was routinely removed after 6 months. After removal, patients must adhere to the prescribed diet. Evaluation, including assessments for nutritional deficits (iron, folic acid, B12 vitamin, and magnesium), was conducted one month after removal. Patients were scheduled for MBS within 4 to 8 weeks after IGB removal. The choice of the best MBS was personalized based on individual characteristics, comorbidities, prior treatments, preferences, and surgeon recommendations. Broadly, RYGB was recommended for individuals with GERD, hiatal hernias or grade C-D esophagitis, and SG was indicated for patients with inflammatory bowel disease and those at high risk for gastric cancer. All MBS were done by laparoscopy in a standardized 5 trocar technique: RYGB was performed with a small gastric pouch, a biliopancreatic limb of 100 cm, and an alimentary limb of 120 cm. Extended biliopancreatic limbs were considered in some cases. SG was calibrated using a 48-Fr Fouchet tube, sectioning the stomach from the gastric antrum (6 cm proximal to pylorus) to 1 cm lateral to the angle of His.

### Clinical Data Evaluated

Several variables were analyzed: age, gender, anthropometric data (weight, height and BMI), previous gastric banding, time with IGB, complications and intolerances during IGB, return visits to the emergency room (ER), readmission rate, early removal of IGB, % total weight Loss (%TWL) with IGB, mortality rate, type of MBS performed, weight and %TWL after MBS (1 year and 2 years), nadir after MBS, and global weight loss, %TWL after both IGB and MBS (1 year and 2 years), and peri-operative complications (conversion to open surgery and major complications within 30 days after surgery). This data was gathered from clinical electronic records.

BMI was defined as weight (kg) / (height x height (m^2^)). The %TWL was calculated by the following formula: [(initial weight − follow-up weight)/initial weight] × 100 [13].

Intolerances to the IGB were defined as excessive vomiting and nausea. Complications during IGB were grouped according to the Clavien-Dindo Classification [14]. Patients readmitted to the hospital were classified as Clavien II. Minor complications included Clavien I and II, while major complications comprised Clavien III, IV, and V.

Suboptimal clinical response of IGB was defined as %TWL < 5%. The presence and improvement of obesity-related comorbidities were also analyzed regarding DM2, HT, dyslipidemia and OSA. Patients with prediabetes (Hb1Ac between 5.7 and 6.4%) were included in the DM2 group. Criteria for improvement followed the guidelines from the American Society for Metabolic and Bariatric Surgery (ASMBS) consensus statement [13]. Optimal clinical response of MBS was defined as %TWL ≥ 20% considering weight before and 2 years after MBS [15]. Nadir weight after MBS was defined as the lowest weight recorded after surgery. Optimal clinical responses of IGB + MBS strategy was defined as %TWL ≥ 25% and BMI < 35 kg/m^2^, considering initial weight before IGB and weight 2 years after surgery.

### Statistical Analysis

Continuous variables with normal distribution were described using mean and standard deviation (SD), while median and interquartile range (IQR) were used for non-normal distribution. Normal distribution of continuous variables was assessed by histogram, Q-Q-Plot, and Shapiro–Wilk test. Categorical variables were described using frequencies and percentages.

A *p* value ≤ 0.05 was considered statistically significant. Statistical analysis with adequate tests (parametric, non-parametric, correlations and crosstabs) were performed using IBM SPSS Statistics 26® (SPPS Inc., Chicago, Ill., USA).

## Results

We identified 74 patients with BMI ≥ 50 kg/m^2^ proposed to IGB placement prior to MBS that met study criteria. Baseline characteristics are presented in Table 1.

**Table 1:** Baseline demographics and clinical characteristics (*n* = 74)

Female sex; *n* (%)	56	(75,7)
Age (years); mean (SD)	43.5	(11,8)
Height (cm); median (IQR)	162.0	(11)
Weight (kg) before IGB; median (IQR)	150.5	(31)
BMI (kg/m^2^); median IQR)	58.8	(8)
Prior gastric band*; n* (%)	5	(6.8)
Revisional surgery*; n* (%)	3	(4.1)
Surgery performed; *n* (%) SG RYGB SADIS	17 54 1	(23.6) (75.0) (1.4)
DM2; (*n/*total)	19	/51
HT (*n/*total)	33	/51
Dyslipidemia; (*n/*total)	19	/51
OSA; (*n/*total)	35	/51

*SD*, standard deviation; *IQR*, Interquartile range; *BMI*, body mass index; *SG*, sleeve gastrectomy; *RYGB*, Roux-en-Y gastric bypass; *SADIS*,* s*ingle anastomosis duodenal-ileal bypass with sleeve; *DM2*, type 2 diabetes mellitus; *HT*, hypertension; *OSA*, obstructive sleep apnea

Intragastric balloon outcomes are presented in Table 2. Following IGB, the mean %TWL was 14.2% (SD 8.5), with a wide range (minimum =  − 0.7%; maximum = 33.8%). Ten (13.5%) patients achieved a %TWL < 5%, which means optimal clinical response in 86.5% patients after IGB. There were no statistically significant differences in %TWL between female (mean 14.1%, SD 9.2) and male patients (mean 14.5%, SD 6.1) (*p* = 0.84) after IGB.

**Table 2:** IGB outcomes and complications (*n* = 74)

Time with IGB (days); median (IQR)	203	(39)
Early removal; *n* (%)	4	(5.4)
Weight lost with IGB (kg); mean (SD)	22.2	(13.4)
%TWL; mean (SD)	14.2	(8.5)
% TWL minimum – maximum	− 0.7	to 33.8
% TWL > 5 (optimal clinical response); *n* (%)	64	(86.5)
Clavien-Dindo Classification of complications; *n* (%)	16	(21.6)
I	3	
II	6	
IIIa	3	
IIIb	0	
IVa	2	
IVb	1	
V	1	

*SD*, standard deviation; *IQR*, interquartile range; *%TWL*, percentage of total weight loss; *IGB*, intragastric balloon

There were 16 (21.6%) complications associated with IGB, mainly classified as Clavien I or II. Of the 9 minor complications documented, we must note: (1) a patient diagnosed with mild acute pancreatitis and (2) a patient treated for Grade D Esophagitis. Furthermore, 4 (5.4%) major complications were recorded: (1) one death due to respiratory complications immediately after IGB placement, specifically aspiration pneumonia progressing to acute respiratory distress syndrome and refractory septic shock; (2) gastric distention and perforation 5 months after IGB placement, requiring urgent laparotomy and intensive care treatment due to septic shock-related organ dysfunction; (3) a severe propofol allergic reaction during IGB placement, leading to cardiac arrest and multi-organ dysfunction, progressing to intestinal ischemia and requiring urgent laparotomy; and (4) a respiratory insufficiency with acidemia, after IGB placement, requiring treatment in the Intensive Care Unit with non-invasive ventilation.

The median time between IGB removal and surgery was 36.5 days (IQR 109). RYGB was performed in 54 (75%) patients. All surgeries were performed laparoscopically without any conversion to open surgery, and no MBS-related mortality was recorded. There were no major complications detected within 30 days after surgery. Minor complications and nutritional deficiencies were not analyzed, as they were beyond the scope of this study.

Mean %TWL 2-years after MBS was 27.8% (SD 12.6). After surgery, mean nadir weight was 93.9 kgs (SD 19.8) and median maximum weight loss was 37.0 kgs (IQR 20.8).

Overall results of IGB followed by MBS are presented in Table 3. Fifty-one patients were suitable for analysis at 2 years of follow-up, representing 71,8% of the initial sample. One year after MBS, the overall mean %TWL was 39.5% (SD 13.2), and after 2 years, it was 38.2% (SD 11.6). After 2 years, the relative mean contribution of IGB to total weight loss was 37% (SD 21.9), while MBS accounted for 63% (SD 22.9). Optimal clinical response with IGB + MBS (%TWL ≥ 25% and BMI < 35 kg/m^2^), at 2-year follow-up, was achieved by 23 (45.1%) patients, with 11 (21.6%) patients reaching a BMI below 30 kg/m^2^.

**Table 3:** Weight loss and comorbidities outcomes

	Initial	After IGB	1 year after MBS	2 years after MBS
Weight (kg); median (IQR)	150.5 (31,0)	130.5 (31.5)	95 (32)	94 (32)
BMI (kg/m^2^); median (IQR)	58.8 (8)	50.9 (9.8)	37.2 (10.4)	37.9 (11.2)
%TWL; mean (SD)		14.2 (8.5)	28.9 (13.7)	27.8 (12.6)
DM2; *n*/total	19/51	19/51	14/51	9/51
HT; *n/*total	33/51	34/51	24/51	24/51
Dyslipidemia; *n/*total	19/51	25/51	22/51	19/51
OSA; *n/*total	35/51	29/51	13/50	10/51

*SD*, standard deviation; *IQR*, interquartile range; *IGB*, intragastric balloon; *MBS*, metabolic and bariatric surgery; *TWL*, total weight loss; *%TWL*, percentage of total weight loss; *DM2*, type 2 diabetes mellitus; *HT*, hypertension; *OSA*, obstructive sleep apnea

Comparative analysis between IGB complications and outcomes after MBS are presented in Table 4. The studied variables did not reveal statistical correlation with the incidence of IGB complications, meaning that IGB complications did not appear to have a statistically significant impact on the overall %TWL post-IGB + MBS. Two patients were absent from comorbidities resolution study due to the aforementioned complications.

**Table 4:** Statistical analysis of potential factors influencing IGB complications and impact on outcomes

	Patients with IGB complications (*n* = 16)	Patients without IGB complications (*n* = 58)	*p*-value
Age (years); mean (SD)	44.4 (10.6)	43.2 (12.2)	0.71^*^
Initial Weight (kg); median (IQR)	149.5 (19)	151.0 (33)	0.82^**^
Previous gastric band; *n* (%)	1 (6.3)	4 (6.9)	1.0^†^
Revisional surgery; *n* (%)	0	3 (5.2)	1.0^†^
%TWL 1-year global after IGB + MBS; mean (SD)	40.3 (11.2)	39.2 (13.8)	0.81^*^
%TWL 2-year global after IGB + MBS; mean (SD)	39.7 (8.9)	37.8 (12.3)	0.64^*^
%TWL > 5 after IGB and %TWL > 20 2 years after MBS; *n/*total (%)	6/11 (54.5)	27/40 (67.5)	0.43^††^

*SD*, standard deviation; *IQR*, interquartile range; *IGB*, intragastric balloon; *MBS*, metabolic bariatric surgery; *TWL*, total weight loss; *%TWL*, percentage of total weight loss

^*^Independent samples T-Student test

^**^Independent samples Mann–Whitney

^†^Fisher’s exact test

^††^Chi-square test

There was a statistically significant correlation between post-surgery nadir weight and weight loss after IGB (*p* = 0.025), but the coefficient of determination was only 6.8%, indicating low association strength.

Achieving %TWL > 20% after MBS, considering weight loss only after surgery, was not statistically associated (*p* = 0.50 – chi-square) with a %TWL > 5% after IGB.

## Discussion

Our study suggests that IGB, used as a “neoadjuvant” treatment before MBS in patients with BMI ≥ 50 kg/m^2^, resulted in acceptable weight loss (mean %TWL 14.2%) but with high variability between patients. When considering IGB optimal clinical response as %TWL ≥ 5%, the majority (86.5%) of patients achieved these results. It was suggested that %TWL above 5% will benefit patients, namely with relevant improvements in cardiovascular risk factors [16].

Intra-gastric balloon prior to MBS in patients with a BMI above 50 kg/m^2^ may be used to reduce intra-operative difficulties and complications. In our series, there were no cases of post-operative mortality, conversion to open surgery, or major complications within 30 days after MBS. While additional data such as operative time, estimated intra-operative blood loss, or subjective assessments of difficulty by the surgical and anesthesiology teams could provide further insights, the low peri-operative complication rate after MBS would require a large sample of patients to detect significant differences.

Outcomes after IGB did not predict results after MBS: significant weight loss after surgery was achievable even in patients that did not experience substantial weight loss after IGB. As the mechanisms inducing weight loss differ significantly between these two treatments, IGB should not be used as a “trial” for selecting patients who will achieve better results after MBS. Patients who either decline or are not candidates to MBS after IGB have a high risk of recurrent weight gain in the long-term follow-up [17], suggesting that IGB has a limited efficacy as a single treatment [18], especially in patients with higher BMI. According to the guidelines of the *American Association of Clinical Endocrinologists and American College of Endocrinology*, MBS must be considered only after exhausting conservative approaches [19]. While IGB is presumed to be more effective than nutritional and lifestyle changes for weight loss and improving obesity-related diseases [20], there is limited evidence when comparing IGB to recent anti-obesity medication for the treatment of patients with BMI above 50 kg/m^2^.

Additional studies have suggested that hospital readmission due to intolerances or complications related to IGB were associated with suboptimal weight loss outcomes [21]. However, our study found no weight loss significant differences between patients with and without IGB complications. In fact, individuals facing complications exhibited a higher %TWL, but caution is warranted due to the wide standard deviation. This result may be attributed to the fact that nausea and vomiting were considered minor complications in our study, and patients experiencing these symptoms might probably lose more weight.

The mean %TWL was 14.2% after IGB, consistent with other studies [22, 23]. It is important to note the significant variability in weight loss after IGB, averaging 20 kg during this phase, as other studies have consistently demonstrated inferior weight loss outcomes [21, 24].

Although MBS accounts for the majority of %TWL (61.35%), about one-third is attributed to IGB. However, greater weight loss during IGB does not consistently lead to superior outcomes after MBS. Global weight loss outcomes, 2 years after MBS, appear similar regardless of the relative distribution of weight loss during each phase (IGB vs MBS).

Intra-gastric balloon exhibited a non-negligible complication rate of 21.6%, most of them minor complications, emphasizing the importance of careful consideration in patient therapeutic planning. Consistent with prior research, most patients experienced nausea, abdominal pain, and vomiting. These symptoms are considered common, typically arise shortly after IGB placement and persist for a few days, reflecting the gastric adaptation to the foreign body [25, 26]. Other pre-identified complications were GERD, gastric ulcers, and balloon migration. More serious and rare events were small bowel obstruction, perforation, and death [27]. In our study, a patient initially presenting dyspepsia in the ER was subsequently diagnosed with acute pancreatitis, a previously reported IGB complication [28]. Another patient was treated for Class D Esophagitis, a condition frequently observed in IGB with lower filling volumes [29].

In our series, 6 patients were previously submitted to adjustable gastric band, which is considered a relative contraindication for IGB placement [30]. Although this could indicate an increased risk, our study showed no additional IGB complications in this small subset of patients.

Early IGB removal occurred in 5.4% of patients, mainly due to refractory vomiting. The documented death resulted from respiratory complications post-IGB placement, progressing to aspiration pneumonia, consistent with other reports [31]. We must also note other major complications related to general anesthesia, and a case of gastric distension and perforation, a severe yet rare complication of IGB [32].

New devices aim to reduce associated complications. One such approach is the swallowable nitrogen-mix gas-filled balloon, which has been shown to potentially double weight loss compared to lifestyle therapy alone, while demonstrating a low incidence of major complications [33]. Another innovative option is a swallowable and self-degradable balloon designed to empty autonomously after 4–5 months without complications [34]. These devices may reduce complications associated with IGB and reignite the interest of IGB in the treatment of obesity, particularly in patients with BMI ≥ 50 kg/m^2^. Nevertheless, the absence of pre-procedural endoscopic examination may hide anatomical abnormalities and mucosal lesions, potentially leading to other complications during treatment [34].

In terms of comorbidities, our study only revealed the resolution of OSA in 7 patients after IGB. Improvements in DM2, HT and dyslipidemia were not observed. In contrast, other studies [31] reported major improvements in associated comorbidities. A meta-analysis concluded that IGB may reduce %Hb1Ac by 0.62%, with similar effects as antidiabetic medications and a significant reduction in systolic blood pressure was also documented, surpassing the impact of weight loss medications [35]. Regarding dyslipidemia, Shah, R.H., et al. concluded that, while IGB provides some benefits, it is not as effective as anti-obesity medications [35].

After MBS, there was significant improvement in comorbidities. Several studies have demonstrated MBS effectiveness in improving HT, DM2, and dyslipidemia [36]. The metabolic effect of MBS has a different role compared to the mechanical effect of the IGB.

Despite the presented two-step strategy for patients with Obesity IV/V, only 21.6% achieved a final BMI below 30 kg/m^2^, indicating that roughly 1/5 of patients will achieve non-obese status. This prompts reconsideration of treatment objectives for Obesity IV/V and suggests the need for alternative or sequential therapies like pharmacotherapy or more aggressive surgeries for this difficult subset of patients.

Recently, new anti-obesity medication is challenging the current indication for IGB in patients with higher BMI. These medications achieve good weight loss outcomes, high rates of comorbidity improvements, and few complications. Although there is limited data about the outcomes of anti-obesity medication in patients with a BMI above 50 kg/m^2^ before MBS, this strategy is being adopted in many centers. Presently, access to these medications can be hindered by costs and availability. Further studies are warranted to compare outcomes of anti-obesity medication with other treatments for patients with obesity grade IV and V.

Our study had some limitations due to the retrospective design and small sample size from a single center, which may limit the generalization of these findings to other populations.

## Conclusion

Intragastric balloon is a treatment option for patients with BMI ≥ 50 kg/m^2^ before MBS, resulting in acceptable weight loss, but with a non-negligible complication rate that requires careful consideration when planning therapeutic strategy.

Our findings indicate that IGB has minimal impact on comorbidity resolution and weight loss outcomes after IGB do not seem to predict outcomes after MBS.

Only a minority of patients reached a non-obese status 2-year after IGB and MBS, which highlights the need for further treatment options in this difficult subset of patients with BMI above 50 kg/m^2^.

## Data Availability

All the data supporting the findings of this study are available from the authors upon reasonable request.
